# Trivalvular nonbacterial thrombotic endocarditis in a patient with colon adenocarcinoma: a case report

**DOI:** 10.1186/s13256-023-04070-1

**Published:** 2023-08-07

**Authors:** Abdolhamid Bagheri, Mohammad Khani, Tooba Akbari, Erfan Ghadirzadeh, Elham Charkazi, Parastoo Ghorbani

**Affiliations:** 1 Department of Cardiology, Cardiovascular Research Center, Shahid Beheshti University of Medical Sciences, P.O. Box 1998734383, Tehran, Iran; 2 Cardiovascular Research Center, Mazandaran University of Medical Sciences, Sari, Iran; 3 Semnan University of Medical Sciences, Semnan, Iran

**Keywords:** Noninfective endocarditis, Nonbacterial thrombotic endocarditis, Colorectal neoplasm, Case report, Complication

## Abstract

**Background:**

Nonbacterial thrombotic endocarditis is a rare complication of prothrombotic states such as neoplasms that can cause valvular dysfunction and life-threatening complications. Nonbacterial thrombotic endocarditis usually affects the left-sided valves; however, only a minority of cases involving the tricuspid valve have been reported in medical literature.

**Case presentation:**

The current report describes trivalvular involvement by nonbacterial thrombotic endocarditis in a 54-year-old Azeri female patient with metastatic colorectal carcinoma. This case underlines the necessity of evaluating nonbacterial thrombotic endocarditis as a possible consequence in cancer patients. When thromboembolic events are found in the presence of a hypercoagulable state (such as malignancy) and no growth on blood cultures, nonbacterial thrombotic endocarditis could be suspected as the cause.

**Conclusion:**

It is critical to achieve early diagnosis in such a setting to initiate treatment plans and prevent further complications rapidly.

## Introduction

Nonbacterial thrombotic endocarditis (NBTE) is a rare condition defined by aseptic vegetations on the valve leaflets that could cause systemic embolization [1]. It is a rare complication of prothrombotic states such as advanced neoplasms and systemic lupus erythematosus (SLE) [2]. Higher rates of NBTE have been reported in patients with an advanced underlying malignancy compared with the general population (1.25% versus 0.2%) [3]. Adenocarcinomas (for example, of the pancreas, lung, prostate, etc.) are more likely to have been impacted by NBTE compared with other malignancies (2.7% versus 0.47%) [3].

A definitive diagnosis is reached based on histopathological assessment of the surgically removed specimen; however, this is not a common approach [4]. Most clinicians base the diagnosis of NBTE on the observation of valvular vegetation in the absence of imaging evidence of inflammatory damage of the heart valves or any evidence supporting a systemic infection in patients at high risk for NBTE [4, 5]. NBTE usually affects the left-sided valves; however, only a minority of cases involving the tricuspid valve have been reported in medical literature [6]. The current report describes trivalvular involvement by NBTE in a patient with metastatic colon adenocarcinoma. This case underlines the need for clinicians to evaluate NBTE as a possible consequence in cancer patients and to be aware of the uncommon incidence of NBTE in the tricuspid valve.

## Case presentation

A 54-year-old Azeri female with colon adenocarcinoma was referred due to a recent proximal deep venous thrombosis and dyspnea on exertion function class II. The patient had been diagnosed with metastatic adenocarcinoma of the colon and had started on a modified FOLFOX6 chemotherapy regimen 3 months prior without surgical interventions. She did not mention any previous history of cardiac or pulmonary diseases and had a normal psychological background; however, she had a suspicious history of cancer in her mother. She was from a low socioeconomic society and complied poorly with therapeutic and diagnostic approaches.

In vital signs, she had a blood pressure of 136 on 85, a pulse rate of 76, a respiratory rate of 17, an O_2_ saturation of 98%, and a 37.1 °C axillary temperature. On physical examination, significant edema of the left leg was discovered, auscultation of the lungs was clear, and no thrills were felt in palpation; however, a 2/6 early-diastolic murmur was heard on the left lower sternal border, which was not new. She had a normal white blood cell count (7900/μL, ref value: 4500–11,000), anemia (serum hemoglobin: 7.3 g/dL, ref value: 12.1–15.1), normal platelet count (171 × 10^3^/μL, ref value: 150–450 × 10^3^), elevated d-dimer (36,400 ng/mL, ref value: 220–500), normal creatinine (Cr = 1, ref value: 0.5–1.1 mg/dL), and elevated C-reactive protein level (5.26 mg/dL, ref value: 0.3–1) in laboratory tests. Blood culture was also negative.

**Fig. 1:**
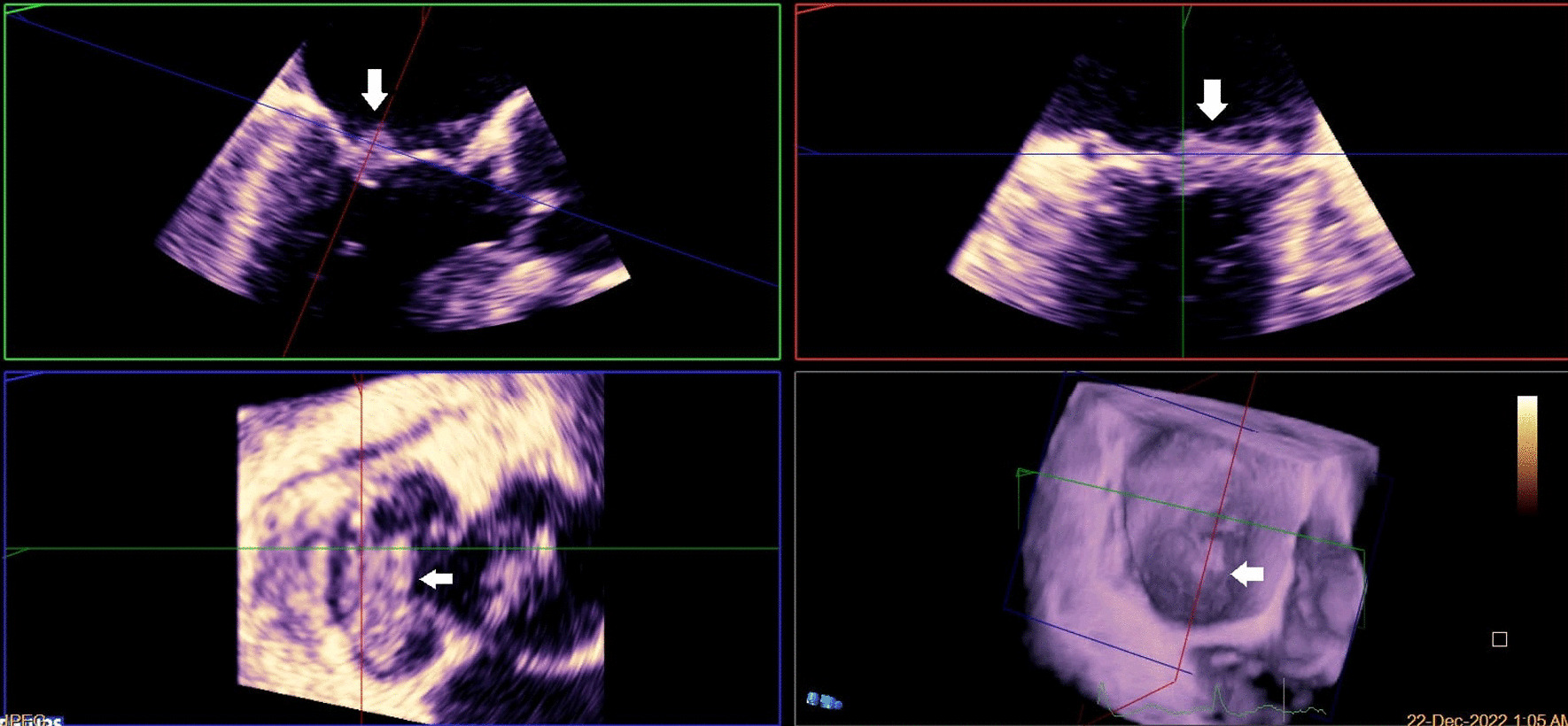
Multiplane reconstruction of MV in three-dimensional (3D) TEE data: vegetation on the A2 scallop (arrows highlighting the vegetation)

**Fig. 2:**
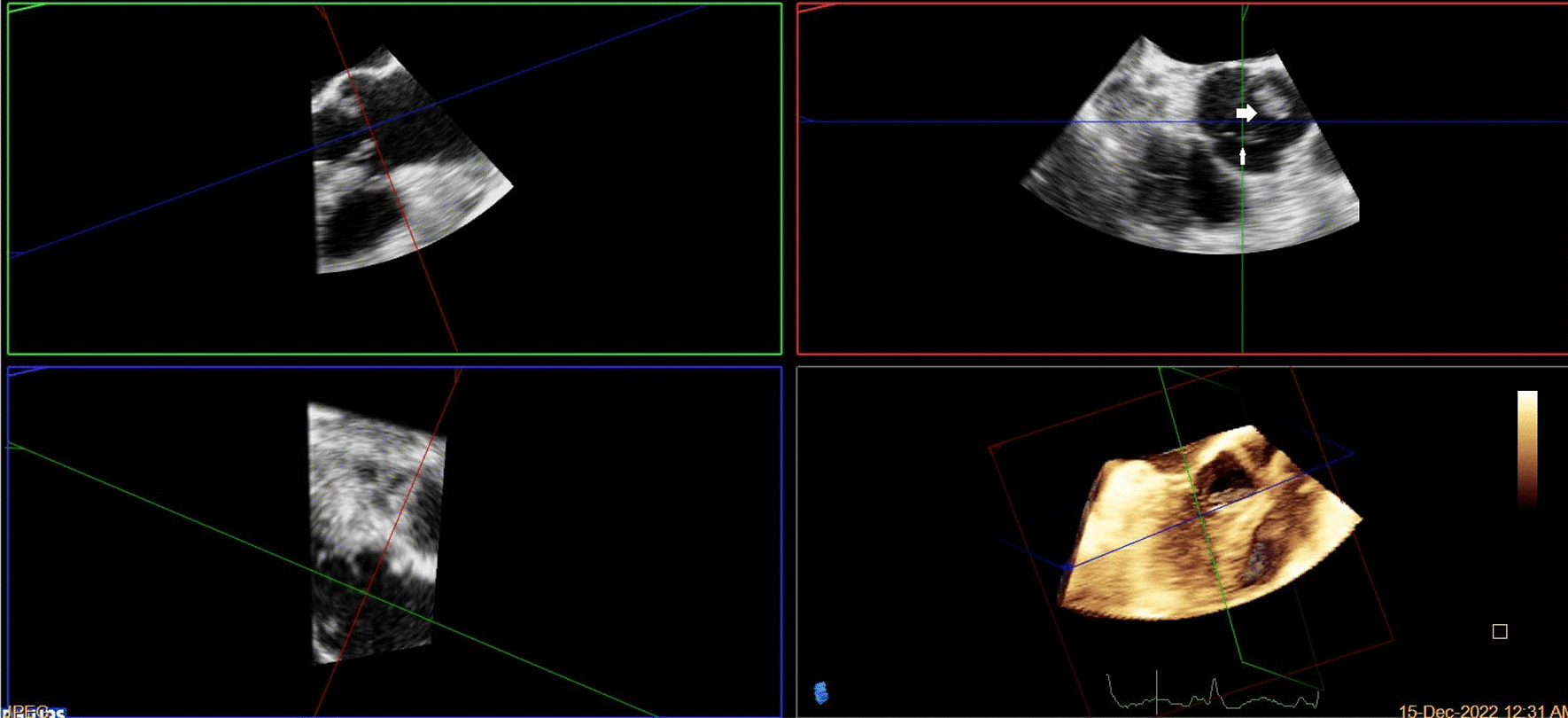
Multiplane reconstruction of AV in 3D TEE data: vegetations were seen on the tip of LCC and RCC

**Fig. 3.**
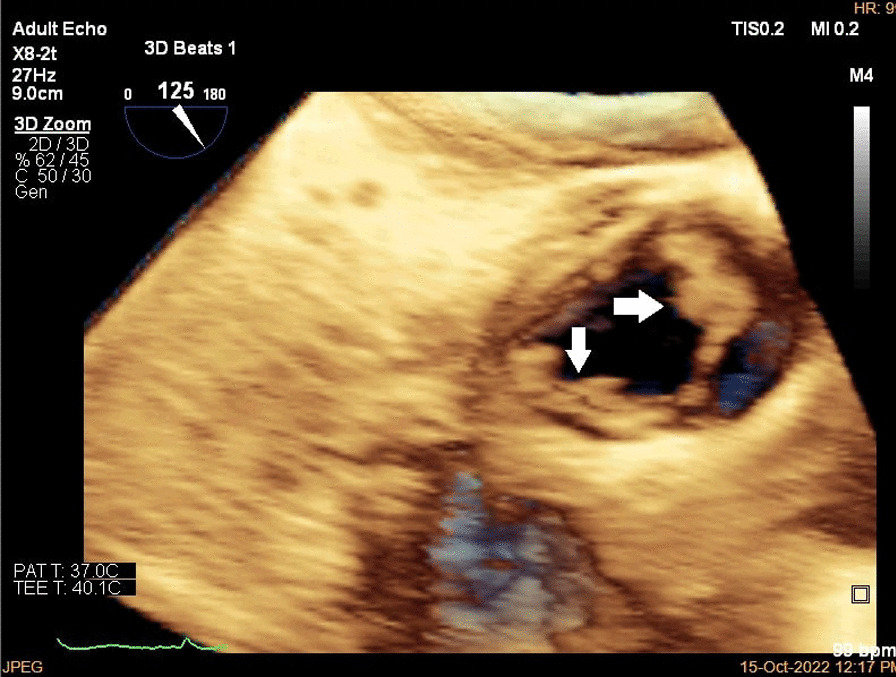
3D zoom from AV in TEE (arrows highlighting the vegetation)

**Fig. 4:**
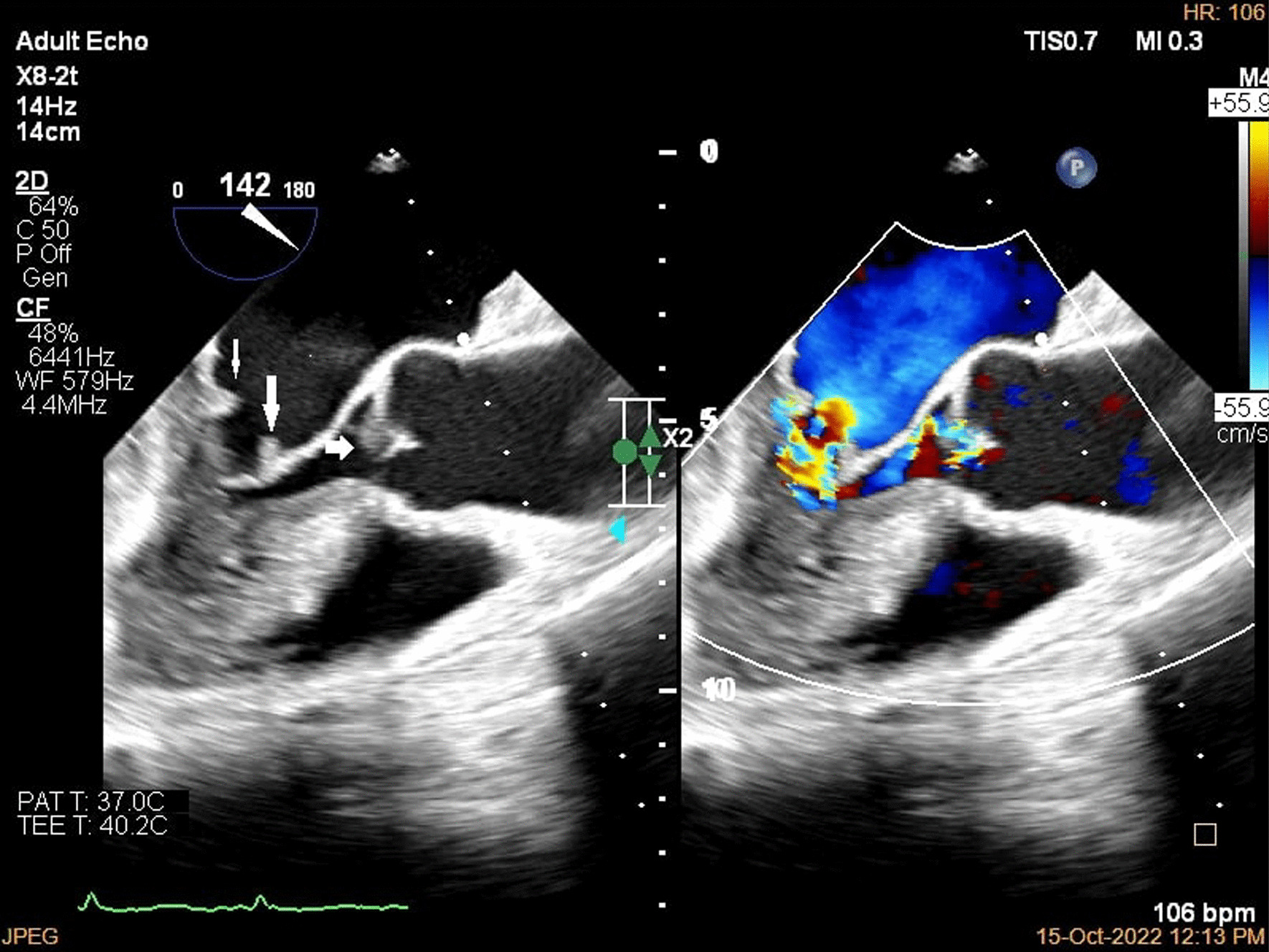
Two-dimensional (2D) TEE in long axis view in diastole; color study revealed mild to moderate aortic insufficiency

**Fig. 5.**
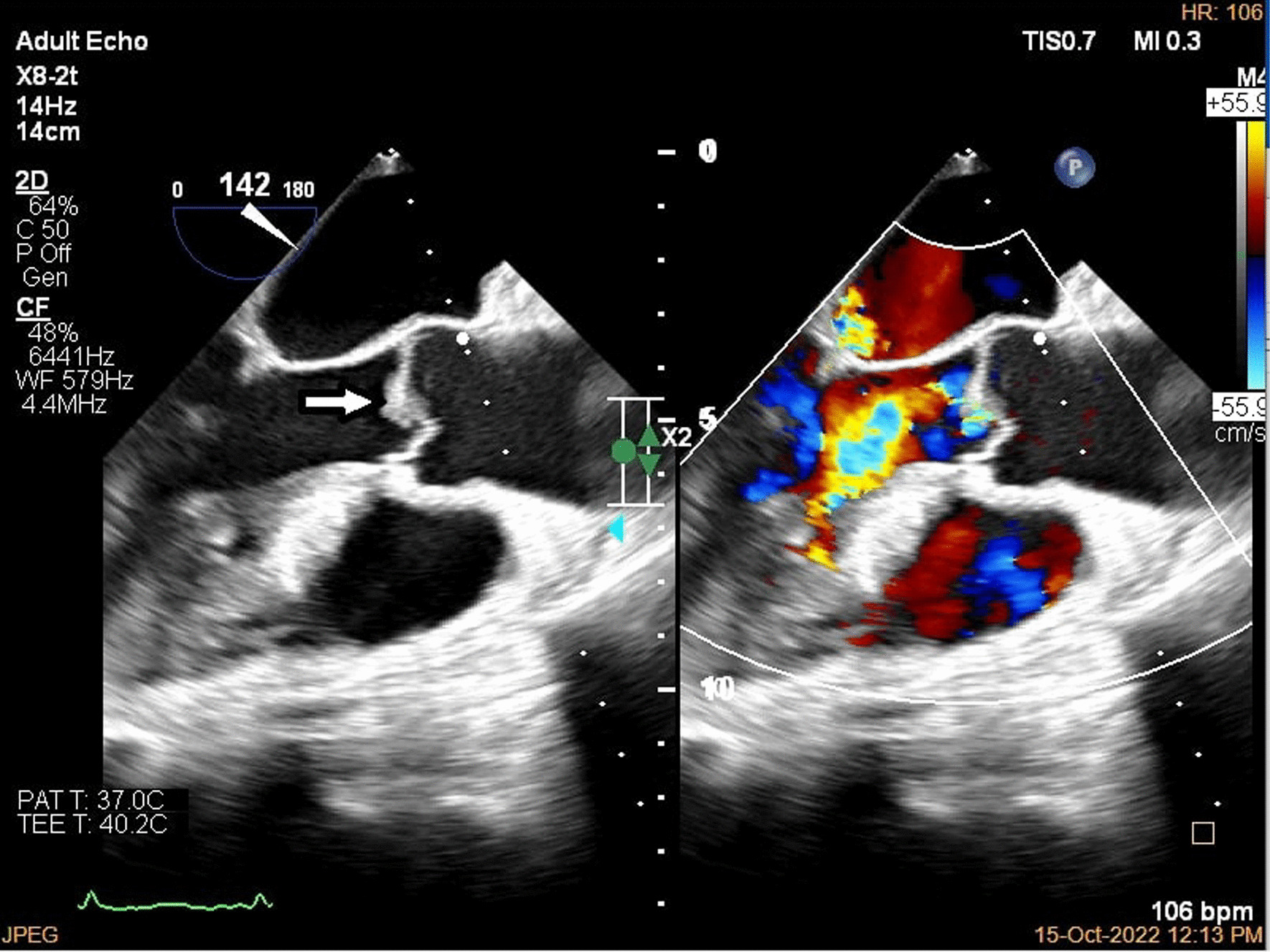
2D TEE in long axis view in systole; color study revealed mild to moderate mitral regurgitation

**Fig. 6.**
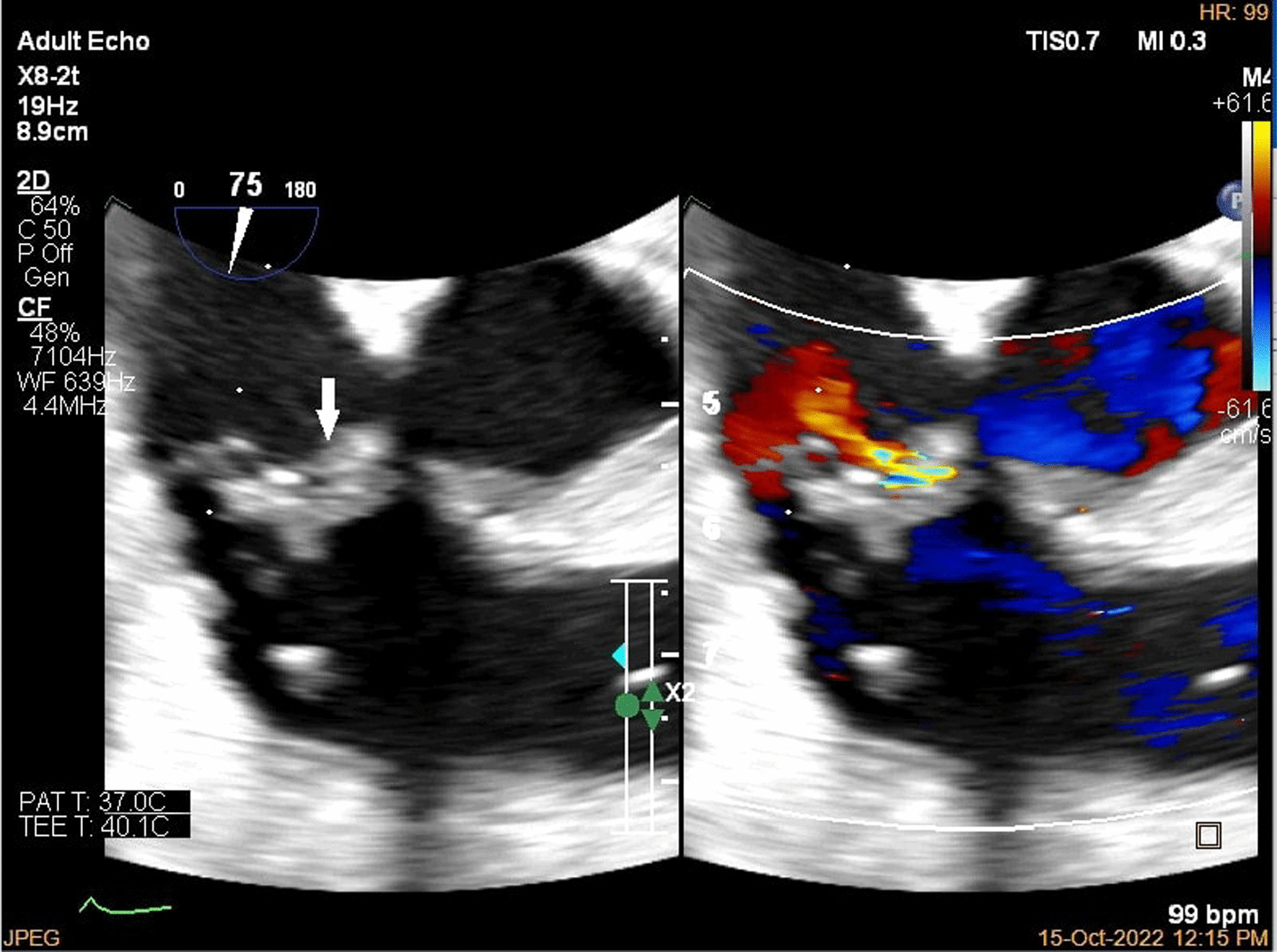
2D TEE in short axis view, vegetation was seen on tricuspid valve leaflet, color study revealed mild to moderate tricuspid regurgitation (arrow highlighting the vegetations)

A brain computed tomography (CT) scan revealed no hemorrhages or ischemia. Pulmonary CT angiography revealed a thrombus in the pulmonary artery of the left lower lobe. Transthoracic echocardiography (TTE) indicated vegetations on the mitral, aortic, and tricuspid cardiac valves, as verified by transesophageal echocardiography (TEE) (Figs. 1, 2, 3). TEE was performed to evaluate valvular structure and function in detail. TEE revealed semimobile echo densities attached to the atrial side of the anterior and posterior mitral leaflet tips with dimensions of 5.7 mm × 3.3 mm and 4 mm × 3.6 mm, respectively, with no destruction and mild to moderate mitral regurgitation. The aortic valve showed two sessile echogenic masses on the ventricular side of the edge of the left coronary cusp (LCC) (9.5 mm × 4 mm) and right coronary cusp (RCC) (6 mm × 4 mm). Tricuspid valve leaflets also showed a fixed 6.4 mm × 2.3 mm echo density with myocardial texture on the anterior leaflet. Other findings included mild tricuspid regurgitation, mild aortic insufficiency, and normal ventricular size and function. Remarkably, despite the abundant vegetation, we found few signs of valve injury and nonsignificant valvular regurgitation (Figs. 4, 5, 6).

Additionally, patients with colon adenocarcinoma are more prone to *Streptococcus bovis* endocarditis; however, *S. bovis* infective endocarditis (IE) could not quickly be ruled out in our patients simply by clinical data owing to modified symptoms and diminished immune system response resulting from chemotherapy and the underlying malignancy [7]. Thus, she was a candidate for more advanced imaging modalities, such as cardiac magnetic resonance imaging (CMR) or positron emission tomography (PET). However, in shared decision-making, she and her family refused to perform advanced diagnostic evaluations. She also refused to initiate antibiotics and further chemotherapy. Finally, our patient was prescribed 1 mg/kg enoxaparin twice a day; however, she could not be monitored owing to her insistence on returning to her village and spending end-of-life time there without medical care. Unfortunately, she died 3 months later due to her advanced metastatic colorectal carcinoma.

## Discussion

NBTE is a rare condition that is frequently underdiagnosed, despite being a dangerous manifestation of cancer-associated hypercoagulability because it is typically asymptomatic unless embolization occurs [8]. NBTE vegetations are aseptic, more fragile, and more prone to systemic embolization than infectious lesions [9, 10]. Anatomically, the mitral valve (MV) is the most commonly affected, followed by the aortic valve (AV) [11]. Nevertheless, involvement of the right-side cardiac valves, such as pulmonary and tricuspid valves, is relatively rare [9]. Multivalvular NBTE is extremely rare, with only a handful of published cases involving three or all four valves [9, 12, 13].

The pathogenesis of NBTE has not been fully identified; however, the interaction between injured endothelial cells resulting from the production of cytokines (such as tumor necrosis factor or interleukin 1 and 6) in the setting of malignancy could activate platelet deposition, particularly in a hypercoagulable state [2, 14]. The macrophage–tumor cell interaction may also overactivate the clotting cascade, resulting in worsening hypercoagulability [8]. A new or altered murmur may warn a clinician of NBTE; however, cardiac murmurs are not common in NBTE and do not have enough sensitivity or specificity to differentiate them from IE [8, 15, 16]. Murmurs frequently exhibit limited reliability as a sign due to the diminutive dimensions of marantic vegetation, alongside the fact that approximately 82% of the affected valves demonstrate no discernible structural impairments [8, 17].

Nevertheless, IE is the major differential diagnosis of NBTE, which should carefully be ruled out by cultures, especially in patients with previous antibiotic exposure [1, 15, 16]. Typically, many clinicians perform thorough assessments of complete blood counts and a minimum of three blood cultures, in addition to a polymerase chain reaction and serologic tests where obtainable, to confirm a diagnosis of NBTE [8]. Additionally, if a specific underlying etiology is not suspected, thorough workup for SLE, antiphospholipid syndrome, and possible malignancy should be conducted [18, 19]. McKay and Wahler introduced a diagnostic criterion for NBTE, consisting of a clinical triad encompassing a murmur, history of multiple systemic emboli, and history of malignancy; however, additional indicators for NBTE include the absence of clinical improvement following treatment for IE, negative results in blood cultures, and history of cerebral embolism with unknown cause [20]. To visualize valvular vegetations, TTE can be helpful; however, TEE should be performed in suitable candidates if TTE is unrevealing.

Nevertheless, neither TTE nor TEE can differentiate between IE and NBTE [4, 11, 19]. In cases where clinical suspicion remains elevated despite a negative TEE, CMR techniques such as fast imaging steady state precession (True FISP), gradient echo, or diffusion-weighted magnetic resonance imaging (MRI) can be employed. Additionally, diffusion-weighted MRI holds promise in differentiating NBTE from IE by assessing the heterogeneous nature of the vegetation [21].

The treatment of NBTE mainly includes managing the underlying medical condition that provokes the formation of vegetation on the valve [8]. In the case of a patient with colon adenocarcinoma, treatment would involve managing the cancer through surgery, chemotherapy, or radiation therapy. Additionally, owing to the fragile nature of the vegetation, the patient would benefit from receiving life-long anticoagulation therapy to prevent the development of further thrombi or the embolization of existing thrombi; however, a brain CT scan should be performed prior to anticoagulant initiation to rule out ischemia or hemorrhage [8, 19, 22]. Heparin-based components, in the form of either unfractionated heparin (UFH) or low-molecular-weight heparin (LMWH), should be utilized for this purpose [19, 22]. These medications have been reported to be more effective in reducing the recurrence of embolization than warfarin. The presence of malignancy can lead to diminished responsiveness to warfarin, potentially owing to the activation of various factors, such as cytokines (interleukins 1 and 6), cyclooxygenase-2 genes, type 1 plasminogen activator inhibitor, cysteine proteases, and tissue factors; however, guidelines support using warfarin over UFH or LMWH in autoimmune and inflammatory disorders owing to extended survival [19, 22, 23]. Anticoagulation should be carried out unless heparin-induced thrombocytopenia or lethal bleeding occurs [19, 22].

In some cases, surgical intervention may be needed, primarily to prevent recurrent embolization where cost‒benefit is favorable [24, 25]. In such circumstances, vegetation can be excised with the preservation of valves (in contrast to IE surgical management), or damaged valves could be surgically replaced [25]. Additionally, anticoagulation therapy should be continued postoperatively when feasible.

Regarding patient outcomes, formally assessing the prognosis of nonbacterial thrombotic endocarditis (NBTE) remains limited in the existing medical literature. However, insights drawn from clinical experience and retrospective studies indicate a bleak outlook, even with anticoagulation therapy. This unfavorable prognosis is attributed to the significant correlation between NBTE and advanced malignancy [11].

Overall, the treatment of NBTE valves involves a multidisciplinary approach comprising a team of healthcare specialists, including cardiologists, hemato-oncologists, and cardiothoracic surgeons, to control the underlying problem and prevent disease-related consequences.

## Conclusion

When thromboembolic events are observed alongside a hypercoagulable state, particularly in cases involving malignancy or SLE, with no growth on at least three blood cultures and no evidence of possible infection in CBC or vital signs, it is essential to consider the possibility of NBTE as the underlying cause. Early NBTE detection is crucial to establish a diagnosis and promptly initiate appropriate treatment strategies. Doing so makes it possible to prevent additional harm to the heart valves and minimize the risk of systemic embolization. Additionally, the therapeutic management of NBTE consists of managing the underlying disease, anticoagulation, and surgical interventions where appropriate. In addition, there is a gap in the medical literature regarding the prognosis of NBTE in different malignancies, which could be the foundation of future studies.

## Data Availability

The data are available with the corresponding author and can be reached on request.
